# Taking pleasure seriously: Should alcohol research say more about fun?

**DOI:** 10.1111/add.16747

**Published:** 2025-01-09

**Authors:** James Nicholls, Geoffrey Hunt

**Affiliations:** ^1^ Institute for Social Marketing and Health, Faculty of Health and Sports Science University of Stirling Stirling UK; ^2^ Centre for Alcohol and Drug Research Aarhus University Aarhus Denmark; ^3^ Institute for Scientific Analysis Alameda California USA

**Keywords:** alcohol, ethics, intoxication, pleasure, public health, risk

## Abstract

**Background:**

This paper invites discussion on whether pleasure should receive more attention in public health‐oriented research on alcohol. While there is a history of sociological and anthropological literature exploring alcohol and pleasure, this is much less common in public health‐oriented alcohol research, and associated advocacy.

**Argument:**

We propose three broad reasons why more extensive engagement with issues of pleasure may be important for public health‐oriented research. The first is epistemological: because overlooking pleasure risks leaving a gap in knowledge of a key component of, and motive for, drinking. The second is ethical: because the prioritisation of long‐term health over shorter‐term pleasures is not uncontested, and needs to be explicitly justified. The third is pragmatic: because ceding the discourse on pleasure to other actors (including commercial ones) risks undermining effective engagement with target populations.

**Conclusions:**

There is strong case for more attention to pleasure in public health‐oriented alcohol research. Key to this is the further development of interdisciplinary perspectives and mixed‐methods research. This brings both conceptual and methodological challenges, many of which remain unresolved; however, bringing these issues to the surface may enable greater clarity on both normative principles (including arguments *against* research engaging with pleasure) and practical questions concerning the design of research and analysis in this area.

## INTRODUCTION

Alcohol research has a problem with pleasure. On the one hand, pleasure is a difficult phenomenon to research, at least from an epidemiological or clinical perspective. On the other, because of its predominating focus on harms, public health‐oriented alcohol research and advocacy can appear to find pleasure problematic in the moral sense. Although most people drink because they enjoy it, much public health discourse downplays pleasure as either marginally significant or as a kind of misperception driven by external forces including marketing, custom, social norms and peer pressure.

This article argues that more attention should be paid to pleasure for three broad reasons. Epistemologically, because understanding pleasure is necessary for any field claiming expertise in drinking behaviours; ethically, because the case for valuing long‐term health over short term pleasure is not self‐evident and needs to be justified; and pragmatically, because currently the discourse on pleasure is largely ceded to those actors (e.g. the alcohol industry) against whom much public health advocacy is directed. We suggest this will require two broad actions: the exploration of novel methods, and interdisciplinary engagement, to better define and measure pleasure; and a sustained debate on how much value (if any) should be accorded to pleasure in public health discourse.

## BACKGROUND

We are not the first to argue for more attention to this issue [1, 2, 3, 4, 5, 6]. Anthropological and socio‐historical research has long recognised pleasure as a key component of drinking behaviours [7, 8, 9, 10], and several sociological studies have explored the complex socio‐cultural factors that shape experiences of pleasure and the relationship between alcohol intoxication and both desire and the performance of social roles [11, 12, 13, 14]. Behavioural psychology has also explored pleasure. In particular, drinking motives research identifies ‘enhancement’ as both a primary driver for consuming alcohol and a primary effect of drinking. Importantly, this research attempts to operationalise pleasure in questionnaires, such as the Drinking Motivations Questionnaire‐Revised [15, 16, 17].

Several papers in the drug field have applied critical perspectives to the study of pleasure and intoxication. Special editions of the *International Journal of Drug Policy* in 2008 and 2017, as well as articles by Hunt and Evans [18] and Ritter [19], have considered the exclusion of pleasure in drug research, and the practical and ethical implications of marginalising pleasure in policy discourse (see Hunt *et al*. [20]). Nevertheless, in relation to alcohol, we would contend that Coveney and Bunton's [2] observation still holds: that whereas ‘a great deal of public health research and practice deals tangentially with issues of pleasure, there have been few attempts to focus directly on the topic’. A search for *Addiction* articles with ‘pleasure’ in the title brings up only six results, two of which are book reviews [19, 21, 22, 23, 24, 25]. On this measure, the long history of socio‐cultural research on this issue seems to have had little impact on public health thinking.

It has been argued that failing to engage with pleasure can undermine the impact of health messaging [26, 27, 28], although this is difficult to test empirically. Beyond this lies a question of principle: does public health research have a responsibility to engage with pleasure when claiming expertise on alcohol consumption? If so, what might a more sustained and sophisticated discussion look like? Debates on alcohol policy often fall into an unhelpful dichotomy with libertarians portraying public health advocates as joyless nannies [29], and control advocates characterising their opponents as either deploying ‘industry arguments’ or betraying a false consciousness sustained by the power of alcohol marketing [30, 31]. The reality is more complex, and a more extensive discussion of pleasure in public health discourse, supported where possible by empirical research, would better reflect this.

Clearly, exploring the attitudes of drinkers, the phenomenology of pleasure and the social construction of intoxicated experiences lends itself to the sociological gaze more than to epidemiology or clinical medicine. However, other barriers exist. Researchers may view pleasure as ‘too frivolous’ [2], ‘too difficult, too slippery’ [32] or ‘too disreputable, too unscientific, to merit systematic and sustained attention’ [33]. Impact prioritisation in research may lead to a focus on harm, but also a perceived ‘professional risk’ of not appearing to take harms seriously [34]. Alcohol researchers may also fear that acknowledging pleasure is viewed as promoting ‘industry arguments’ that strategically undermine public health advocacy [30]. We wish to invite reflection on whether these barriers need addressing, and if so how that might be achieved.

## ALCOHOL, INTOXICATION AND PLEASURE

Alcohol is an intoxicant, and for many drinkers intoxication—to varying degrees of intensity—is pleasurable. Other pleasures are associated with drinking, including taste, the expression of cultural capital and the bonding effects of social ritual [35, 36, 37]. People also drink for reasons beyond enjoyment including: routine, boredom, coping, relaxation or dependency [15, 16, 38, 39, 40]. None of what follows is to suggest the harmful effects of drinking should be downplayed or that there is not a complex relationship between pleasure‐seeking and pain‐avoidance which can drive problematic consumption. It is only that the ‘pleasure’ side of this relationship could be attended to more carefully. Here, we focus on intoxication‐as‐pleasure because some level of intoxication is common in all drinking. These effects vary in quality and intensity along the blood alcohol concentration (BAC) curve [41], but creating a categorical distinction between consumption that is and is not intoxicating elides this reality.

It has long been recognised that intoxication is not simply a matter of subjective experience, but is also socially determined [2, 5, 7, 14, 42, 43, 44, 45, 46, 47, 48]. We not only learn how to drink and take drugs, but intoxicated experiences are shaped by historical and cultural representations, peer group norms and so forth [49, 50]. Intoxicated pleasure is, to some degree, learnt [7], and likely changes in nature over the course of a drinking career (especially if dependence becomes a significant factor). Therefore, understanding intoxication‐as‐pleasure requires engagement with social, cultural and historical factors that shape both expectancies and how intoxicated experiences are interpreted, as well as psychological and pharmacological approaches. Such engagement is necessarily interdisciplinary.

Drinking motives research finds that ‘enhancement’ (drinking because it is ‘exciting’, ‘pleasant’, ‘fun’ etc.) is, alongside ‘coping, the most commonly reported motive for drinking [15, 17]. It is also associated with heavier drinking in both the short and medium term [51]. There are obvious consequentialist reasons to be concerned with this from a health perspective. However, rules‐based (or ‘deontological’) values are also at play in how we think about short term, sensory enjoyment: what is sometimes defined as ‘hedonic’ pleasure (in contrast to the ‘eudaimonic’ happiness achieved through the realisation of ones potential over time [52]). The pursuit of hedonic pleasure is problematized in many ethical, philosophical and religious traditions, and for many reasons. Importantly, contemporary attitudes are shaped not only by long‐standing religious scepticism toward hedonism, but also by a tradition of post‐enlightenment liberalism in which hedonic pleasure is associated with irrationality and irresponsibility [53]. This matters because, within this tradition key ideals such as freedom, agency and moral responsibility are assumed to rest on the capacity for reason [3, 54, 55, 56, 57]. Intoxication presents an unusual ethical problem in this context [3, 58, 59, 60].

We live in a culture that both encourages and condemns the pursuit of hedonic pleasures, and it is within this culture that public health operates. The paradox of ‘neoliberal’ culture is that the pursuit of hedonic experience is both promoted (because the economy demands consumption) and disciplined (because we are required to be productive citizens) [5, 34, 48, 57, 61, 62, 63, 64, 65]. Indeed, Winlow and Hall [66] argue that pleasure‐seeking is best understood as a ‘labour’ of late capitalism. Therefore, analysing intoxication‐as‐pleasure involves not just empirical observation, because it also engages notions of value, identity, rationality, autonomy, moral responsibility and the expression of socio‐cultural capital in a range of different contexts [2, 4, 67].

## DOES PLEASURE HAVE VALUE?

Determining the public good is different to identifying drinking motives. It requires us to ask not whether drinking is simply enjoyable or reinforcing, but whether intoxication has any social, ethical or welfare value: what is the good (if any) of intoxication, and what is the value (if any) of intoxication‐as‐pleasure? Does pleasure need rational and instrumental purposes to be viewed as legitimate [68]? Insofar as public health is liable to be sceptical of hedonic pleasure, it is liable to not ascribe value in this way [49, 69].

Throughout its history alcohol control advocacy has tended to assume that the pleasure of intoxication can simply be disregarded for the purposes of calculating public policy. As one temperance advocate wrote in 1841: ‘[A]bstractly considered, giving up inebriating drinks, involves no sacrifice, because by so doing, we lose nothing; and there can be no sacrifice, where nothing is sacrificed or lost’ [70].

Claims that there is ‘no safe level’ of alcohol affirm something similar. If the value of pleasure is stripped from the equation, or if consumption is framed in medicalised terms, then arguments for restrictions in the service of health appear self‐evident. A recent *Lancet* editorial went further, stating that the mere fact drinking was culturally embedded in many traditions did not ‘justify its continuation’ as a social practice: thus setting out the unusual proposition that, in the case of alcohol consumption, the burden of ethical justification should fall on the existing cultural practice, not on those wishing to restrict it [31].

Arguably, however, public health discourse does not simply devalue pleasure. Rather it asserts a preference for one type of pleasure over another. If ‘eudaimonic’ pleasure is taken to be objectively more valuable than hedonic, then the purpose of reducing alcohol consumption is not ‘taking the fun out of life [but] giving people the precious gift of time to live their lives’ [71]. Health promotion, from this perspective, is justified because it increases longevity, and therefore the temporal capacity to experience happiness. Writing in defence of public health positions on alcohol and pleasure, Daube [72] argued alcohol policy interventions that infringe on other personal liberties are justified because: ‘First, a healthy lifestyle by definition promotes greater capacity to enjoy life, and to enjoy the pleasures life has to offer. Second, the longer one lives, the more opportunities one has to derive enjoyment.’

Although this statement accords with the wider prioritisation of health in modern, affluent societies [73], it nonetheless implies unstated ‘utility preferences’, which are not universally held. The validity of the first assertion may be challenged by what could be called the ‘Keith Richards’ problem—that whereas healthy lifestyles may often correlate to a happier life, it is neither a universal experience nor perception that they do. The latter assertion is also open to question. Longevity undoubtedly creates more opportunities for enjoyment; however, a pessimist may add that it also creates more opportunities for pain, illness and disappointment.

From an equity perspective, it may also be argued that for people experiencing multiple disadvantages, preferring the immediate gains of short‐term pleasure may be rational if future happiness is uncertain because of the other effects of poverty and discrimination. In other words, the expected value of long‐term health may be partly determined by present privilege. The balance between pleasure and pain over a lifetime is not fixed or predictable, and trading uncertain long‐term benefits for short term pleasure is not unusual, nor self‐evidently wrong [55]. Furthermore, for many people who use substances pleasure and risk are not distinct but overlapping, because risk‐taking itself can form a key component of the experience [74, 75, 76, 77, 78].

Public health risks appearing bemused as to why anyone would ever want to drink or get drunk, explaining away pleasure‐seeking motivations by reference to ‘semi‐coercive discourses of “peer group pressure” and “advertising pressure”’ [3] or drinking being ‘portrayed as … fun’ by the alcohol industry [31]. Here, the ‘expert story’ of alcohol as ‘inherently dangerous, being intoxicating, gradually addictive and damaging to the brain and the body’ runs hard against a public view of drinking as generally pleasurable and a ‘positive personal experience’ [79]. Room [1] observes that early survey data gave the impression that ‘all drunkenness in America happened only by accident, as a miscalculation’. However, that is evidently not the case. As Keane [4] argues, if ‘health discourse understands intoxication as an expendable harm with no redeeming qualities, it will be unable to recognise its attractions as anything other than evidence of individual or cultural pathology’. Treating the pleasure of intoxication as if it were a pathology, with no authentic value for drinkers, risks further distancing health advocacy from the lived experience of many whom it addresses [76].

## TALKING ABOUT PLEASURE

In 2016, while discussing newly published low‐risk drinking guidelines, the then Chief Medical Officer for England, Dame Sally Davis, commented that she hoped drinkers would ‘Do as I do when I reach for my glass of wine and think “Do I want my glass of wine, or do I want to raise my risk of breast cancer?”’ [80]. While this may have been an attempt to address pleasure in relation to risk, it betrayed a problematic assumption that ordinary drinkers see that balance in simplistic and dichotomous terms. The comment provoked a backlash in parts of the media, with Davis later saying her framing was a mistake [81, 82, 83, 84]. In 2023, the release of new low‐risk guidelines in Canada, which defined more than two standard drinks per week as increasing risk, provoked some similar commentary, with even the British Broadcasting Corporation describing the recommendations as ‘drastic’ [85, 86, 87]. Arguably, some of the nuance of the guidelines was lost in media translation [86]. However, the response also reflected the fact that for many drinkers restricting consumption to two drinks a week to avoid small absolute risks to health was simply not a serious proposition.

The alcohol industry clearly has reasons to encourage antagonism toward drinking guidelines, but it is not useful to see that as the whole story. The tension between framing alcohol as a source of harm and the experience of it as a source of pleasure requires negotiation. For many, low‐risk drinking guidelines ‘are not much fun at all’ [1], and can appear to show ‘the determined obtuseness of public health when it addresses the risks and pleasures of everyday life’ [4]. If small increases in absolute risk, or drinking above very low levels, are presented as justification for significant policy change, then people may be more easily persuaded that upstream measures are disproportionate. Fitzgerald *et al*. [79] have recently shown that framing communications around information deficits (e.g. lack of health information on labels) or decentring alcohol, rather than dismissing its attractions outright, may be more persuasive in growing support for restrictive policies.

On the other hand, the long declining trend in youth consumption in many high‐income countries suggests public health messaging may have been effective in those settings. Perhaps focusing on risk and harm has fostered conformity with the principle that ‘disciplined pleasure’ [2] is preferable to the riskier pursuit of short‐term fun [88, 89]. However, this remains speculative, and it remains axiomatic in alcohol control advocacy that marketing does increase youth consumption [30]. On this point, accepting that pleasure is an authentic motive for, and consequence of, alcohol intoxication, but that this is also culturally influenced, creates space for nuanced research into marketing effects. Rather than simply looking for associations between exposure and consumption, we may wish to further explore how cultural discourses around alcohol determine how the pleasures of consumption are imagined and experienced. Research building on critical media studies, attending to audience interpretation and the social construction of communication, might provide further insights into how target populations ‘read’ and respond to both alcohol marketing alcohol‐related health messages.

It could be argued that, precisely because the alcohol industry expends enormous energies in reinforcing the association between alcohol and pleasure, public health should leave fun out of things. Perhaps, while it is the job of history and social science to address underlying social phenomena, the job of public health is to defend a value system that asserts the primacy of health over other social goods and to provide a robust counterweight to commercial actors. If we take this dialectical approach, then maybe public health has very good reasons not to talk about pleasure. However, incomplete analyses of social phenomena risk creating unintended policy consequences. Silence on the pleasures that motivate behaviours not only risk causing health messages to fall on deaf ears, but because such silence is ‘not a neutral absence, but rather political in its effects’ [47] it risks entrenching resistance to a health discourse perceived as opposed to fun on principle. Addressing populations across class and other cultural boundaries requires understanding how those communities think about and experience intoxication and pleasure and where those experiences fit into their everyday lives. Marginalising pleasure also risks missing a vital dimension of the subject on which alcohol research and advocacy claims expertise, while reinforcing a puritanical stereotype that makes it easier for the alcohol industry to dismiss its claims, monopolise the discourse of pleasure and so maintain business as usual.

## MOVING FORWARD

Addressing pleasure and intoxication raises difficult methodological issues [52]. However, although these challenges are not easy to resolve there are opportunities for public health research to build on psychological and sociological studies to better understand the phenomenology of intoxicated pleasure, the different ways in which value is ascribed to it by drinkers and the degree to which the experience of pleasure is culturally situated. Developing a more sophisticated understanding of pleasure as a complex set of interrelated feelings and experiences would further support research into what drives behaviours and, by extension, how they might be changed. It could also help public health actors engaged in policy discourse avoid appearing to hold naïve views of pleasure (such as that it is merely an illusory consequence of marketing or that it operates in a narrowly oppositional relationship with health) that do not accord with lived experience.

Lessons and methods from economics could also be more widely applied. For instance, the finding by Amlung *et al*. [90] that the marginal utility of alcohol increased with intoxication (i.e. consumers were willing to pay more the more they consumed) seems important, not least for developing realistic estimates of price elasticities. Utility values accorded to drinking should not be disregarded when assessing economic impacts, because they are a concrete measure of the value accorded to short term pleasure by the consumers about whom public health is concerned. Calculating the utility value of pleasure in relation to the value of harms caused would allow for more sophisticated estimates of costs to society, as well as potential benefits from measures such as tax increases.

There are clearly challenges in developing meaningful epidemiological measures for pleasure. However, the operationalisation of pleasure in existing surveys provides pointers for future development, as do recent experiments in methods such as ecological momentary assessment of mood [39, 40, 91]. Bond and Ford [92], discussing sexual health, argue that epidemiology should address these challenges if a more complete understanding of behaviours and risks is to be obtained. The same principle applies for alcohol.

Hunt and colleagues [93] argue that bridging sociology and epidemiology requires better integration across disciplines, rather than new quantitative tools. Of course, in progressing better or broader interdisciplinary research the onus should not fall solely on public health. There is also an opportunity for those working in disciplines, including psychology, that have explored this topic more deeply to seek further engagement with public health or to make their findings more obviously applicable to public health policy discourse, where that is appropriate. Generally, further development of mixed‐methods approaches will be important. In other cases, simply considering pleasure more routinely in discussion sections, even if only noting it as a missing element, may help provide balance. However, for this to happen pleasure must be viewed as more than a marginal concern and discussion of it treated less as dangerously close to ‘industry’ framing.

We also need more explicit discussion of the ethical principles that prioritise the cultivation of long‐term health over other preferences. What is the underpinning justification for minimising the value of short‐term, potentially risky, pleasures that may, in other respects, strengthen social bonds, create novel experiences or simply feel good in the service of sensible self‐discipline? On what grounds should policies that enforce this trade‐off be supported? There clearly are strong arguments as to why, but they often remain assumed or uncritically deployed (with counter‐arguments dismissed as industry positions or squeamishness toward the ‘nanny state’). Although the task of public health research may be to identify ‘what works’, there remains a responsibility to articulate why its social goals are preferred—especially where public policy is involved.

The problem of how to deal with pleasure in alcohol research is not new, but we feel it bears further reflection. We offer these thoughts because unresolved questions of both principle and practice would, we believe, benefit from further debate within the research and advocacy communities.

## AUTHOR CONTRIBUTIONS

**James Nicholls:** Conceptualization; writing—original draft; writing—review and editing. **Geoffrey Hunt:** Conceptualization; writing—original draft.

## DECLARATION OF INTERESTS

None.

## Data Availability

Data sharing is not applicable to this article as no new data were created or analyzed in this study.
